# Gut Microbiota Regulates the Interaction between Diet and Genetics to Influence Glucose Tolerance †

**DOI:** 10.3390/medicines8070034

**Published:** 2021-07-01

**Authors:** Jeralyn J. Franson, Julianne H. Grose, Kaitlyn W. Larson, Laura C. Bridgewater

**Affiliations:** Department of Microbiology and Molecular Biology, Brigham Young University, Provo, UT 84602, USA; jer.franson@gmail.com (J.J.F.); kaitlynwilliams20@gmail.com (K.W.L.)

**Keywords:** gut microbiome, diet, insulin resistance, glucose tolerance, obesity, triglyceride, high fat, high sugar, leaky gut, PAS kinase, claudin-1

## Abstract

**Background:** Metabolic phenotypes are the result of an intricate interplay between multiple factors, including diet, genotype, and the gut microbiome. Per–Arnt–Sim (PAS) kinase is a nutrient-sensing serine/threonine kinase, whose absence (PASK^−/−^) protects against triglyceride accumulation, insulin resistance, and weight gain on a high-fat diet; conditions that are associated with dysbiosis of the gut microbiome. **Methods:** Herein, we report the metabolic effects of the interplay of diet (high fat high sugar, HFHS), genotype (PASK^−/−^), and microbiome (16S sequencing). **Results:** Microbiome analysis identified a diet-induced, genotype-independent forked shift, with two discrete clusters of HFHS mice having increased beta and decreased alpha diversity. A “lower” cluster contained elevated levels of *Firmicutes, Bacteroidetes, Actinobacteria, Proteobacteria* and *Defferibacteres*, and was associated with increased weight gain, glucose intolerance, triglyceride accumulation, and decreased claudin-1 expression. Genotypic effects were observed within the clusters, lower cluster PASK^−/−^ mice displayed increased weight gain and decreased triglyceride accumulation, whereas upper PASK^−/−^ were resistant to decreased claudin-1. **Conclusions:** These results confirm previous reports that PAS kinase deficiency can protect mice against the deleterious effects of diet, and they suggest that microbiome imbalances can override protection. In addition, these results support a healthy diet for beneficial microbiome maintenance and suggest microbial culprits associated with metabolic disease.

## 1. Introduction

In healthy individuals, increased blood glucose levels trigger beta cells in the pancreas to produce insulin. Extracellular insulin can subsequently bind to insulin receptors on cellular membranes [1], allowing for increased glucose uptake by the cell, through translocating glucose transporters to the cellular membrane and stimulating glycogen synthesis [2]. A decrease in the cell’s sensitivity to insulin is known as insulin resistance, which can lead to hyperglycemia, hepatic lipid synthesis, and adiposity [3]. The severity of insulin resistance is used to classify individuals as either prediabetic or type 2 diabetic, with prediabetes affecting 33.9% and diabetes affecting 10.5% of adults in the United States (US) [4]. Of adults diagnosed with diabetes, 87.5% are overweight or obese [5], with obesity alone affecting 39.8% of all US adults. Thus, understanding the pathways contributing to these interrelated diseases is essential to the development of proper treatments, as well as preventative strategies. Studies of insulin resistance and obesity have led to the identification of multiple contributing factors, including diet, microbiome, and genetics.

Between 2013 and 2016, 37% of adults in the United States ate fast food, typically high in fat, sugar and calories, on a given day [6]. Adults in the United States consume 14.9% of their daily calories from sugar [7], which can lead to metabolic disease [8], and a high-fat diet is a significant driver in the development of obesity [9]. In addition to over nutrition’s caloric contribution to obesity, the influence of diet on obesity and insulin resistance can be traced through the direct effect that diet has on the gut microbiota. Over 1000 bacterial species have been identified in the human intestine [10], with concentrations ranging from 10^3^ bacterial cells/gram (bacteria/g) in the duodenum, 10^4^ bacteria/g in the jejunum, 10^7^ bacteria/g in the ileum, and 10^12^ bacteria/g in the colon [11]. The colon microbiota benefit the host through the breakdown of otherwise undigestible complex polysaccharides into short-chain fatty acids (SCFA), supplying up to 10% of the host’s daily energy requirements [12,13]. The duodenum, jejunum and ileum have a higher prevalence of Firmicutes (Lactobacillaceae family), Proteobacteria, and Actinobacteria, while the colon has a higher prevalence of Bacteroidetes, Firmicutes (Lachnospiraceae and Ruminococcaceae families), and Verrucomicrobia (*Akkermansia* genus) [11]. This diverse collection of bacteria exists in a delicate dance with the host, as the bacteria are influenced by immune cells and nutrients provided by the host, while the host’s health is subsequently affected by the bacteria in a beneficial or detrimental manner.

The gut microbiota plays an important role in the development of obesity and insulin resistance, which was first shown by Gordon et al. [14]. They reported shifts in the ratio of Bacteroidetes, a Gram-negative bacterial phylum, and Firmicutes, a Gram-positive bacterial phylum, in genetically obese mice. Further studies confirmed a preponderance of Firmicutes in both obese human subjects [15] and high-fat fed mice [16], with a high-fat, high-sugar (HFHS) diet also altering the gut microbiota and increasing intestinal permeability [17]. Additionally, studies showed that transplantation of microbiota from obese human donors into germ-free mice led to the development of weight gain and insulin resistance [18,19,20,21]. Specific strains of bacteria isolated from human hosts and transplanted into germ-free mice were shown to induce the correlated phenotype (either lean or obese) found in the host, including *Enterobacter cloacae* B29 (induced obesity [19]), *Bifidobacterium pseudocatenulatum* C95 (improved hypoglycemia [22]), and *Akkermansia muciniphila* (improved glucose tolerance and body weight [21,23,24]). Germ-free mice on a high-fat diet are protected from obesity and insulin resistance [25], and oral antibiotics ameliorate the effects of a high-fat diet on weight gain, adiposity, glucose intolerance, and inflammation [16]. This effect of the microbiome on weight gain, adiposity, glucose intolerance, and inflammation may in part be due to intestinal permeability. A high-fat diet has been shown to increase intestinal permeability [16] and significantly decrease the levels of tight junction proteins, including claudin-1 [26], which is expressed in the epithelial barrier and plays a crucial role in the regulation of intestinal permeability [27]. Bacterial lipopolysaccharides (LPS), also known as endotoxins, are pro-inflammatory and can pass through the intestinal epithelium into the bloodstream when the tight junctions between epithelial cells are disrupted.

In addition to diet and the microbiome, genetics plays an important role in insulin resistance and obesity, with hundreds of associated variants identified. Herein, we study the interplay of diet and the microbiome with the phenotypes of Per–Arnt–Sim kinase-deficient (PASK^−/−^) mice, namely, resistance to weight gain, adiposity, liver triglyceride accumulation, and insulin resistance when placed on a high-fat diet [28,29,30]. We recently characterized the phenotype of these mice on a western-style HFHS diet, reporting resistance to liver triglyceride accumulation as well as decreased metabolic rate [31]. The effects of PAS kinase on liver triglyceride accumulation have been attributed to the essential role of PAS kinase in SREBP-1 maturation [32], and the possible phosphorylation and regulation of USF1 [33], which are both key transcription factors in the hepatic synthesis of fatty acids and triglycerides. The increased metabolic rate may also be due to USF1, which has been shown to regulate respiration in yeast in a PAS kinase-dependent manner [33]. PAS kinase also plays a role in the development of maturity-onset diabetes of the young, perhaps through its reported regulation of insulin gene expression and glucagon secretion [34,35,36,37,38,39,40,41,42]. PAS kinase has been specifically shown to regulate pancreatic duodenal homeobox-1 (PDX1), which is required for the function of insulin-producing islet beta cells [36,38,40], as well as alter glucose-sensing pathways, such as glucokinase [30].

Despite the clear role of PAS kinase in metabolic health, no studies have been reported on the gut microbiome of PASK^−/−^ mice. Herein, we provide supporting evidence that gut microbiota contributes to metabolic health in response to diet and are able to override genetic influences (PASK^−/−^) to induce altered metabolism and inflammation, including liver triglyceride accumulation, adiposity, insulin resistance, and intestinal permeability. Although all mice on the HFHS diet displayed an expected shift to lower alpha diversity and increased beta diversity on the HFHS diet, a key finding was a remarkable forked separation of the microbiome composition observed in both wild-type and PASK^−/−^ mice on the HFHS diet, which became the focus of our investigation.

## 2. Materials and Methods

Animals: All procedures were carried out with the approval of the Institutional Animal Care and Use Committee (IACUC) of Brigham Young University (protocols 16-1003 and 13-1003). Two PASK^+/−^ female and one PASK^+/−^ male C57BL/6 (Charles River Laboratories Wilmington, MA, USA) mice were generously donated by Jared Rutter (University of Utah) [28] and bred at Brigham Young University to produce a colony. PASK^+/−^ mice from the colony were bred to produce the WT and PASK^−/−^ groups used in this study. Upon weaning at 3 weeks of age, littermates were randomly assigned to either a normal chow diet (NCD) (8604; Tekland Diets, Madison, WI; protein 32% kcal, fat 14%, carbohydrate 54%) or a western-style high-fat, high-sugar diet (HFHS) (D12266Bi; Research Diets, Inc., New Brunswick, NJ; protein 16.8% kcal, fat 31.8%, carbohydrate 51.4%). Mice were co-housed according to sex, genotype, and assigned diet with four cohorts utilized in this study including wild-type on an NCD (WT-NCD), wild-type on an HFHS (WT-HFHS), PASK^−/−^ on an NCD (PASK-NCD), PASK^−/−^ on an HFHS diet (PASK-HFHS). Both male and female mice were utilized at the beginning of the study, with male mice becoming the focus of the remainder of the study due to the pronounced metabolic phenotype previously observed [31]. All mice were housed with no more than five mice per cage, on a 12-h light/dark cycle. Water and food were freely available. PASK genotypes were determined by polymerase chain reaction (PCR) of tail snip genomic DNA specimens using the following primers: PASK for (5′-GAAGTCACCCCCGATCCCCTCCTAAC-3′), PASK MUT rev primer (5′-ACTTTCGGTTCCTCTTCCCATGAATTC-3′), PASK WT rev primer (5′-CTAGCCATGGTGCTTACCCTC-3′).

Glucose tolerance testing (GTT) and insulin tolerance testing (ITT): All mice were fasted 6 h prior to both GTT and ITT, with water freely available. Blood glucose levels were measured using the TRUEresult glucometer (Nipro diagnostics, Fort Lauderdale, FL). An initial blood glucose reading was taken prior to injection. For GTT, a 20% glucose solution in PBS was injected intraperitoneally (IP) at a dose of 1 mg/g body weight. Blood glucose samples were measured at 5, 15, 30, 60, 90 and 120 min after injection. For ITT, 0.375 units/kg body weight of 0.5 U/mL insulin was administered IP (Humulin R; Lilly, Indianapolis, IN, USA). Blood glucose levels were measured at 15, 30, 45, 60, 90, and 120 min after injection. Mice with multiple readings below 20 mg/dL and demonstrating signs of insulin shock were IP injected with 100 µL of a 20% glucose solution in PBS and removed from the analysis. Food was made readily available and the mice were observed for recovery. GTT and ITT tests were staggered at 15, 19 and 23 weeks for GTT, and 16 and 24 weeks for ITT. Area under the curve (AUC) was determined using GraphPad Prism version 8.0.0 (GraphPad Software, San Diego, CA, USA).

Tissue harvest: At 25 weeks of age mice were euthanized by cervical dislocation. Tissues and organs of interest were excised, cleaned, weighed, flash frozen in liquid nitrogen, and stored at −80 °C.

Sample preparation for metagenomic analysis of gut microbiota: To track changes in weight and gut microbial contents, mice were weighed weekly and fecal samples were collected and placed on dry ice until storage at −80 °C. Bacterial DNA was isolated and purified from fecal pellets using the extraction protocol described in Godon et al. [14] with the following changes: samples were homogenized in the Next Advance Bullet Blender Storm (Next Advance, Averill Park, NY, USA), using 3.2 mm stainless steel beads (SSB32; Next Advance, Averill Park, NY, USA), and then cells were disrupted with 0.1 mm glass beads (GB01; Next Advance, Averill Park, NY, USA). After isolation, purified DNA was suspended in 10 mM Tris (pH 8.5) and stored at −20 °C. DNA concentration and purity was estimated by measuring the A260/A280 ratio with a NanoDrop spectrophotometer (Nanodrop Technologies, Wilmington, DE, USA), and integrity of purified DNA was checked using 0.8% agarose gel electrophoresis with ethidium bromide staining.

Library construction: 16S rDNA gene libraries were prepared according to directions by Illumina using AMPure beads (Beckman Coulter Life Sciences, Indianapolis, IN, USA) for PCR cleanup. SequalPrep normalization plates (Invitrogen, Frederick, MD, USA) were used for final DNA normalization of all samples with the exception of the first batch of gut microbiota sequencing on male week 22 mice, where samples were normalized manually by determining the concentration of DNA by NanoDrop spectrophotometry (Nanodrop Technologies, Wilmington, DE, USA), then adjusting it appropriately. Paired-end sequencing was performed on the Illumina Hi-Seq 2500 platform in the BYU DNA Sequencing Center.

Analysis of metagenomic data: 16S rDNA sequences were analyzed using the QIIME2/2017.10. software package [43]. Read joining, denoising, demultiplexing, and feature assignments were accomplished using the Dada2 [44] plug-in. Forward reads were truncated 23 bp to trim amplicon primers. Reverse reads were truncated at 249 and 240 base pairs to insure overlap of reads. Samples from the created BIOM table [45] were then filtered to remove features that appear in less than 2 total samples (singletons), samples that contain less than 10 features, and features not assigned to at least phyla level. Phylogenetic distances were computed using q2-feature-classifier [46] with naïve-Bayes fit [47]. Alpha and beta diversity were calculated using core metrics rarefied to a sampling depth of 8000. Principle coordinate analysis (PCoA) visualizations were created using EMPeror [48,49]. Permutational multivariate analysis of variance (PERMANOVA) [50] was used to compare differences in beta diversity between groups. Alpha diversity was calculated using Faith’s phylogenetic diversity (PD) and Kruskal–Wallis one-way analysis of variance [51,52]. Taxonomy was assigned using q2-feature-classifier plug-in [46] using Greengenes13_8 85% OTUs trained with the following primer sequences: F-CCTACGGGNGGCWGCAG R-GACTACHVGGGTATCTAATCC. A heatmap of changes in relevant bacteria was made using QIIME2/2017.10. Values from the heatmap for statistical analysis were reconstructed using ImageJ software [53].

Triglyceride assays: Mouse liver samples were homogenized in 5% NP-40 substitute water using the Next Advance Bullet Blender Storm (Next Advance, Averill Park, NY, USA) with 0.9–2 mm stainless steel beads (SSB32; Next Advance, Averill Park, NY, USA). Hepatic triglyceride levels were measured using the BioVision (Milpitas, CA, USA) triglyceride quantification colorimetric/fluorometric kit (K622) according to manufacturer’s protocol, and absorbance was measured at 530–590 nm. Protein concentration was determined using the Pierce Coomassie Plus (Bradford) assay reagent (ThermoFisher Scientific, Waltham, MA, USA).

Immunoblotting: Colon tissue samples were lysed in 2X RIPA buffer volume/sample volume, with 10 µL/mL protease and phosphatase inhibitor cocktail (#78440, Thermo Fisher, Rockford, IL, USA). All samples were homogenized using the Bullet Blender Storm 24 (Next Advance, Averill Park, NY, USA), using 0.9–2 mm stainless steel beads. Following homogenization and lysis, samples were centrifuged at 16,000 rcf for 10 min and the supernatant was collected and stored at −80 °C. Protein levels were quantified using the Pierce BCA assay kit (Thermo Fisher, Rockford, IL, USA) and a microplate reader (BioTek, Minooski, VT, USA). Equal amounts of protein from colon lysates were combined with 5× lane marker sample buffer (Thermo Fisher, Rockford, IL, USA), heated in a boiling water bath for 5 min, and then loaded onto a 4–15% SDS-PAGE gradient mini-PROTEAN TGX gel, 15 µL/well volume (Bio-Rad, Hercules, CA, USA) for separation. Multiple runs were averaged (n = 1–5 per sample) for JMP-based statistical analysis.

An internal standard (WT-NCD) was included on every gel for normalization comparison between gels. Semi-dry electrotransfer of proteins to a 0.45 µm nitrocellulose membrane in transfer buffer (20% methanol in tris/glycine buffer) was performed using the Bio-Rad Trans-Blot Turbo (Invitrogen, Carlsbad, CA, USA) mixed MW midi program. After transfer to the nitrocellulose membrane, non-specific proteins were blocked in a 5% milk solution (non-fat dry milk in 1× tris-buffered saline (TBS)) and washed in TBST (0.05% Tween-20 in TBS). The membrane was then incubated overnight with claudin-1 and β-actin primary antibodies at 1:1000 dilution (Cell Signaling, Danvers, MA), diluted in BSA (2.5 g bovine serum albumin in 50 mL TBST). Following overnight incubation and washing, the membrane was then incubated for 60 min under foil with secondary antibodies, 1:10,000 dilution (IRDye 680RD goat/anti-rabbit, IRDye 800CW donkey/anti-mouse LI-COR, Lincoln, NE, USA) in BSA and washed with TBST. Membranes were scanned on the LI-COR reader using default parameters. Protein expression levels were evaluated using the LI-COR imaging software. The resulting readings were then normalized against the WT-NCD internal standard. Any samples in which the loading control was <0.7 or >1.3 relative to the internal standard were discarded.

Statistical Analysis: All data are shown as mean ± SEM using GraphPad Prism 7.0. ANOVA from QIIME2/2017.10 [43] summary data were performed using GraphPad Prism version 8.0.0 (GraphPad Software, San Diego, CA, USA). Factorial ANOVA or ANOVA was performed using JMP Pro version 14 SAS Institute Inc., Cary, NC, 1989–2019 with Tukey’s post hoc test for three-factor and two-factor interaction analysis or Dunnett’s post hoc test for comparison with control (WT-NCD) as indicated. Alpha diversity of microbiota data was analyzed using Kruskal–Wallis one-way ANOVA [51]. Permutational multivariate analysis of variance (PERMANOVA) [50] was used to compare differences in beta diversity between groups. Tertiles were assigned by sorting each group sequentially and dividing the rankings into thirds.

## 3. Results

### 3.1. PASK^−/−^ Deletion Does Not Protect against Weight Gain on an HFHS Diet, but Does Protect against Hepatic Triglyceride Accumulation and Claudin-1 Decrease in Male Mice While Causing Increased RFP in Female HFHS Mice

**Figure 1:**
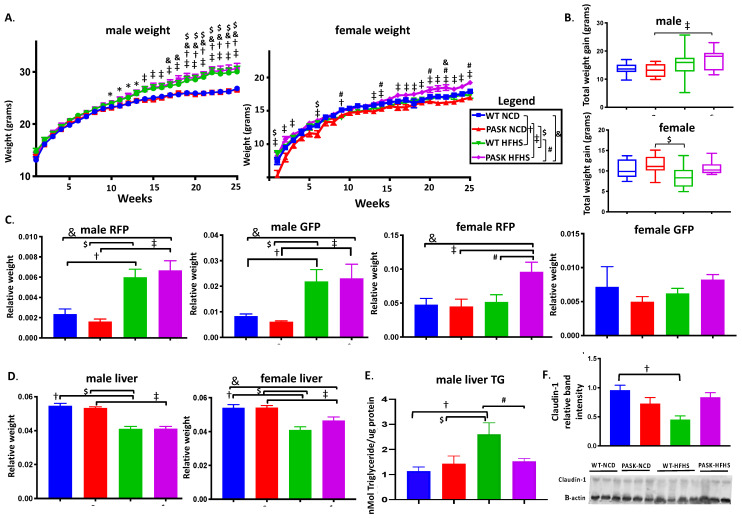
Diet, influenced by genotype, alters body and tissue weight of WT and PASK-deficient (PASK) mice. (**A**) Weekly body weight of male and female mice displays weight gain in response to HFHS diet. (**B**) Total weight gain of male and female mice. (**C**) Relative tissue weights of male and female retroperitoneal fat pad (RFP) and gonadal fat pad (GFP) expressed as tissue weight/final body weight. (**D**) Male and female relative liver weight (tissue weight/final body weight). (**E**) Hepatic triglyceride (TG) accumulation of male mice (*n* = 4–8). (**F**) Representative immunoblots of colon claudin-1 levels *n* = 5–7 per group. B-actin was used as an immunoblot loading control. All data are expressed as mean ± SE. All groups *n* = 13–19 with the exception of male liver TG and claudin-1 immunoblot. Factorial ANOVA (JMP Pro version 14) followed by post hoc Tukey HSD statistical analysis was performed on male and female mice individually because statistical differences were observed for both sexes at all time points. *P*-values less than or equal to 0.065 are shown (WT-NCD compared with WT-HFHS **^†^**, NCD compared with PASK-HFHS **^‡^**, WT-HFHS compared with PASK-HFHS ^#^, NCD compared with WT-HFHS **^$^**, WT-NCD compared with PASK-HFHS **^&^**). A corresponding legend is provided in the top panel.

Male PASK^−/−^ mice were previously reported as resistant to weight gain, liver triglyceride accumulation and insulin resistance, when placed on a high-fat diet [28]. In addition, we recently reported protection from triglyceride accumulation in male, but not female, PASK mice on a western-style diet—one high in both fats and sugars (HFHS) [31]—from which this study is an extension. These known phenotypes were further investigated in this study, to compare with microbiome changes. For mice in this study, male WT-HFHS (*p* = 0.022) and PASK-HFHS (*p* = 0.0025 or *p* = 0.0037) both had significantly higher final body weights, as well as weights at most time points after 11 weeks on the HFHS diet when compared to their normal chow diet (NCD) genotype control, or the WT-NCD, respectively (Figure 1A). In addition, female PASK displayed greater weights on the HFHS diet compared to the NCD, despite the smaller weight gains than males, with PASK-HFHS mice having significantly greater weight gains relative to WT-NCD after 16 weeks on the HFHS diet (Figure 1A). Only male PASK mice displayed significant differences between diets in total weight gain, which were once again due to an interaction between both diet and genotype (Figure 1B). When analyzed by factorial ANOVA, these final weight differences for male and female mice were due to an interaction between both the diet and genotype. Despite randomized cohort assignment from multiple litters, the female PASK-NCD mice displayed visibly lower starting weights than the WT-NCD mice; however, they were not significantly different by factorial ANOVA. In addition, when ignoring the NCD mice and comparing HFHS, there are still significant differences between the WT and PASK-HFHS mice at multiple time points. Thus, both male and female PASK-deficient mice were not protected from the HFHS diet, but instead had trending increases in body weight.

The relative weights (tissue weight/final body weight) of male retroperitoneal fat pads (RFP) (WT *p* = 0.0029, PASK *p* = 0.0002) and gonadal fat pads (GFP) (WT *p* = 0.0608, PASK *p* = 0.0141) also displayed differences relative to their NCD genotype controls, due to an interaction between the diet and genotype (Figure 1C). No significant differences were seen when comparing the HFHS groups. In contrast, female mice displayed no significant increase in GFP on the HFHS diet, but PASK-deficient females displayed a two-fold increase in RFP on the HFHS diet, relative to PASK-NCD or WT-NCD controls (*p* = 0.0042 and 0.0141, respectively, Figure 1C). This observation of significant differences in the body weight at certain time points, and the final RFP weight, is the first report of what appears to be dyslipidemia in the female PASK-deficient mice, which have generally shown no reported PASK^−/−^ phenotype, including no observable difference in liver triglycerides [31].

We have previously shown that male, but not female, mice are protected from liver triglyceride accumulation on an HFHS diet [31], and others have shown dramatic protection of male mice on a HF diet [28]. We analyzed both liver weights and male liver triglyceride accumulation. The weight of the livers of both male and female mice were significantly reduced in response to the HFHS diet, and, once again, these effects were due to an interaction between genotype and diet, by factorial ANOVA (Figure 1D). No significant difference was observed between the WT and PASK male or female HFHS mice; however, the weight of the female PASK livers tended to be not as reduced as the WT mice on the HFHS diet (*p* = 0.0001 compared to the WT-NCD, and *p* = 0.0138 compared to PASK-NCD), which is suggestive of a protective effect (Figure 1D). Despite the lack of protection against body weight gain or liver weight reduction by PAS kinase deficiency, male PASK-HFHS mice were protected from liver triglyceride accumulation in response to the HFHS diet, while WT mice displayed elevated triglycerides relative to the WT-NCD control (*p* = 0.0234) (Figure 1E, *p* = 0.0592 between PASK and WT HFHS). We previously reported this protection from triglyceride accumulation of male, but not female, mice in a study of mice from this same cohort [31]. Due to the dramatic body weight gain and liver triglyceride accumulation phenotype in male mice, as well as the previous report of male-specific phenotypes [28,31], male mice were the focus of the remainder of this study.

To better understand the mechanisms contributing to the increased weight gain and decreased hepatic triglycerides seen in PASK^−/−^ mice on the HFHS diet, the protein levels of claudin-1 were measured in colon tissue, to assay for effects on intestinal permeability. Colon claudin-1 expression significantly decreased in WT-HFHS mice compared to WT-NCD mice (Figure 1F, *p* = 0.0080), confirming previous reports of a decrease in claudin-1, and hence increased intestinal permeability in response to a high-sugar diet [27]. As with the hepatic triglyceride accumulation, PASK-HFHS mice displayed resistance to this decrease in claudin-1 expression when compared to WT-HFHS mice (Figure 1F, *p* = 0.0080). Factorial ANOVA confirmed these differences to be due to an interaction between genotype and diet.

### 3.2. PASK Deletion Does Not Significantly Alter Blood Glucose Levels

**Figure 2:**
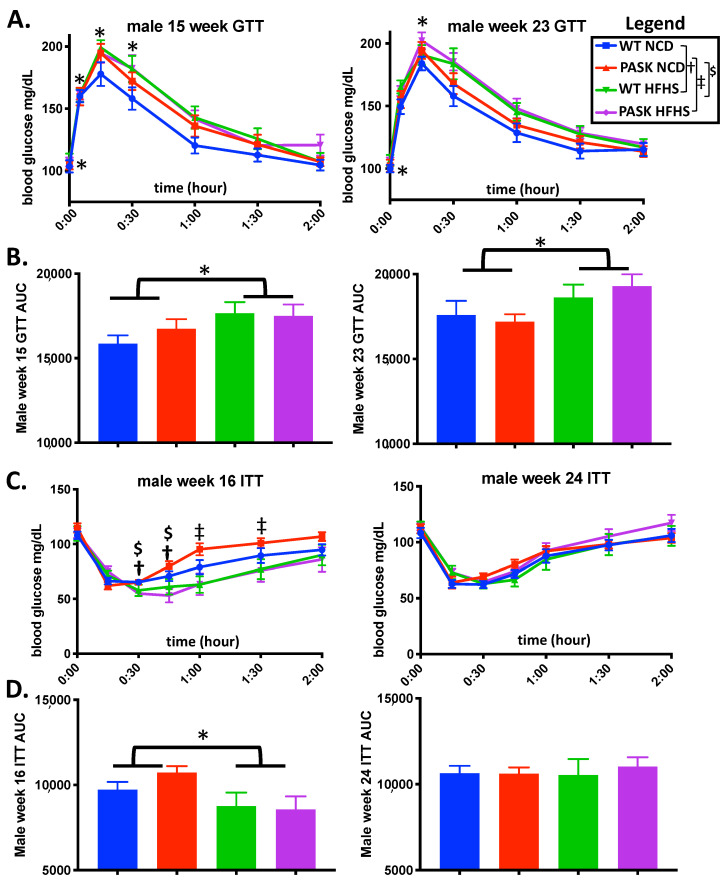
The HFHS diet effects both GTT and ITT response. (**A**) Blood glucose levels during IP glucose tolerance testing (GTT) at 15 weeks and 23 weeks on the HFHS diet. (**B**) Area under the curve (AUC) from (**A**). (**C**) Blood glucose levels during IP insulin tolerance testing (ITT) at 16 weeks and 24 weeks on the HFHS diet. (**D**) Area under the curve (AUC) for (**C**). All data were analyzed by factorial ANOVA in JMP Pro version 14 with post hoc Tukey HSD analysis to investigate interactions between diet and genotype and post hoc Dunnett’s analysis for either diet and genotype separately when no interaction is seen. *P*-values less than or equal to 0.065 are shown (WT-NCD compared with WT-HFHS **^†^**, NCD compared with PASK-HFHS **^‡^**, NCD compared with WT-HFHS **^$^** and * indicates differences due to diet, namely, all mice on NCD versus all mice on HFHS diet). A corresponding legend is provided in the top right. All groups *n* = 7.

Previous research has shown that the deletion of PASK (PASK^−/−^) imparts a protective effect against high-fat diet-induced insulin resistance [28,29]; however, there have been no reports on the study of insulin resistance of these mice on an HFHS diet. A significant difference in glucose response (measured as area under the curve) at 15 and 23 weeks was seen in the combined group of all mice on the HFHS diet, when compared to all mice on the NCD diet (*p* = 0.0414 and 0.0316 for each week, respectively) (Figure 2A,B). However, no significant difference was seen between the WT and the PASK mice on the HFHS diet, suggesting the effects were diet based. Factorial ANOVA also suggested that these results are diet based. However, examining the effects of diet within each genotype separately, by ANOVA, revealed that the diet impaired glucose response for the WT mice (*p* = 0.0420), but not the PASK mice (*p* = 0.3946), at 15 weeks, and the PASK, but not WT, mice displayed a significant difference based on the diet at 23 weeks (PASK *p* = 0.0197, WT *p* = 0.3655). These results may suggest some subtle differences that could be investigated further with larger cohorts. For ITT, the combined mice once again displayed an effect due to the diet at 16 weeks (*p* = 0.0170), with the HFHS diet causing apparent increased sensitivity, and no apparent effect was observed when comparing the HFHS WT and PASK mice to one another (Figure 2C,D). Examining the effects of diet within each genotype separately revealed that the diet significantly impaired insulin response for the PASK mice, but not the WT mice (*p* = 0.0175). No significant differences in ITT were observed in any of the cohorts at 24 weeks (Figure 2C,D). In summary, factorial ANOVA suggests that all observed differences in GTT and ITT are due to diet.

### 3.3. Gut Microbiota Composition Is Determined by an Interaction between Diet and Genotype

**Figure 3:**
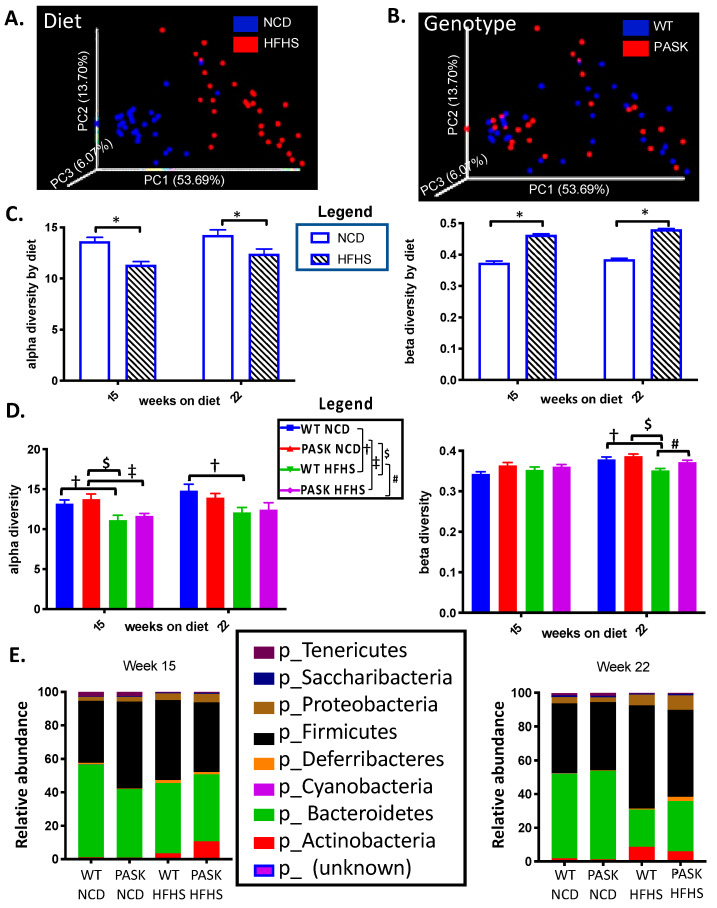
Diet, influenced by genotype, alters gut microbial diversity. (**A**) Comparison of weighted UniFrac principal coordinates analysis (PCoA) distances in microbial diversity at 22 weeks between NCD (blue) and HFHS (red). (**B**) Comparison of weighted UniFrac distances at 22 weeks between WT (blue) and PASK^−/−^ (red). (**C**) Comparison of alpha and beta diversity between NCD and HFHS at 15 weeks (*n* = 21–25) and 22 weeks (*n* = 27–29). (**D**) Comparison of alpha and beta diversity by diet and genotype. (**E**) Relative abundance at phyla level in week 15 samples and week 22 samples. QIIME2/2017.10. software package [43] was used to analyze the microbiome. Alpha diversity was analyzed by Faith’s phylogenetic diversity [52] and is expressed as mean plus SEM, distances to NCD with Kruskal–Wallis follow-up. Beta diversity is expressed as weighted UniFrac [54] mean plus SEM with permutational multivariate analysis of variance (PERMANOVA) [50]. “*” indicates differences due to diet, namely, all mice on NCD versus all mice on HFHS diet (*p* < 0.05). For panel D, factorial ANOVA (JMP Pro version 14) with post hoc Tukey HSD analysis was used to investigate interactions between diet and genotype. *P*-values less than or equal to 0.065 are shown (WT-NCD compared with WT-HFHS **^†^**, PASK-NCD compared with PASK-HFHS **^‡^**, WT-HFHS compared with PASK-HFHS **^#^**, PASK-NCD compared with WT-HFHS **^$^**). A corresponding legend is provided in the top right.

To study the effects of PASK^−/−^ and diet on the gut microbiota, fecal samples, collected at 15 and 22 weeks on the diet, were selected for bacterial DNA isolation and sequencing. Preliminary results of the weighted Unifrac principle coordinate analysis (PCoA) at 22 weeks showed a clear separation by diet, with the NCD mice closely clustered together and the HFHS diet mice displaying a more diverse, altered microbiome (Figure 3A). The genotype did not appear to influence the overall separation of the samples, with both genotypes equally dispersed among the two diet groups (Figure 3B).

Diet played a significant role in shaping the differences in alpha diversity, as measured by Faith’s phylogenetic diversity [52] (15-week *p* = 0.00002 H = 18.2, *q* = 0.0002; 22-week *p* = 0.002 H = 9.55), which showed the microbial diversity within a single group (Figure 3C). The beta diversity, or measure of similarity/dissimilarity between two groups, also altered in response to diet (15-week *p* = 0.001, 22-week *p* = 0.001) (Figure 3C). When the samples were analyzed by factorial ANOVA, for both diet and genotype, the samples taken after 15 weeks showed differences in alpha diversity for both the WT (*p* = 0.064) and PASK (*p* = 0.0273) mice on the HFHS diet, relative to their NCD controls, while the samples at 22 weeks on the diet displayed differences for only the WT mice (*p* = 0.0581). In contrast, no significant differences were observed in the beta diversity, when analyzing for both diet and genotype at 15 weeks, but at week 22 significant differences were observed between WT-HFHS and either of the NCD controls (*p* = 0.0165 for WT and 0.0001 for PASK). In addition, the WT and PASK-HFHS cohorts show significance when compared (*p* = 0.0613). These results suggest that diet played the determinative role in the composition of the gut microbiota; however, the factorial ANOVA indicated that the significant differences are due to an interaction with genotype as well (Figure 3D). When analyzing the relative abundance of the key phyla present at these time points, similar alterations are observed at both the 15- and 22-week time points (Figure 3E). These alterations include increased *Actinobacteria* and *Deferribacteres*, and decreased *Bacteroidetes* in cohorts on the HFHS diet.

### 3.4. Bacterial Composition Is Associated with Weight Gain and Undergoes a Population Fork at Week 22 on the HFHS Diet—A Reanalysis of the Data by Microbiome Composition

**Figure 4:**
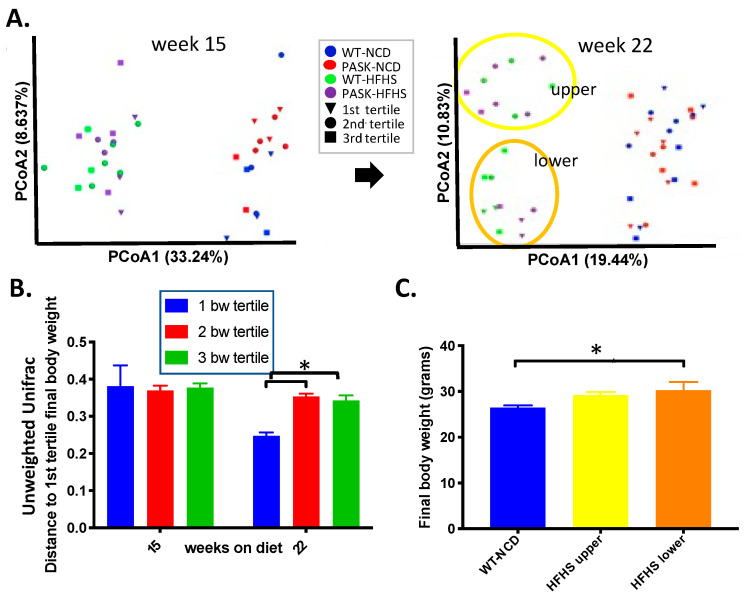
Gut microbial composition is associated with weight gain on an HFHS diet. (**A**) Unweighted UniFrac principal coordinates analysis (PCoA) distances in microbial diversity plot of week 15 samples and week 22 samples coded by final body weight tertile per cohort, with oval outline of the HFHS lower cluster (orange) and upper cluster (yellow). (**B**) Comparison of unweighted UniFrac [54] distances by final body weight tertile of HFHS males at 15 and 22 weeks. (**C**) Final body weights of HFHS mice divided by unweighted UniFrac PCoA cluster compared to the WT-NCD. QIIME2/2017.10. software package [43] was used to analyze the microbiome and produce (**A**). ANOVA was performed from (**B**) summary QIIME2/2017.10. data using GraphPad Prism version 8.0.0 (GraphPad Software, San Diego, CA, USA), and for (**C**), ANOVA in JMP Pro 14, to determine significance followed by post hoc Dunnett’s analysis for comparison with WT-NCD (*p* < 0.05 is indicated by “*”).

To further study the roles diet and microbiota composition played in the development of obesity and glucose intolerance on the HFHS diet, we analyzed unweighted Unifrac principle coordinate analysis (PCoA) results by body weight at the time of microbiome analysis. The mice on an HFHS diet fell into two apparent microbiome groups, termed the upper and lower clusters, at week 22, but not week 15, suggesting the microbiota shift in the HFHS developed over time (Figure 4A, oval outlines at week 22). Note that the shift in beta diversity, observed in Figure 3D, also occurred at week 22. This shift in the microbiome is reflected in the unweighted Unifrac distances between the first and second tertile (*p* = 0.0004), and between the first and third tertile (*p* = 0.0017) of the final body weight in the HFHS week 22 samples, with the heaviest mice (1st tertile, triangles) all belonging in the lower cluster (Figure 4B). Accordingly, the HFHS lower cluster had significantly higher final body weights (Figure 4C) when compared to the WT-NCD (*p* = 0.0478), whereas the HFHS upper cluster did not (*p* = 0.1854). These data suggest the microbiota shift that occurred in a subset of mice on the HFHS diet could be a factor in the development of obesity.

**Figure 5:**
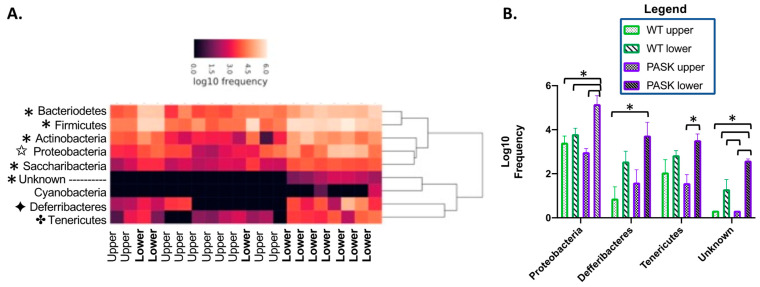
Diet protects against microbiome imbalance. (**A**) Heatmap of week 22 male HFHS samples with upper or lower cluster indicated on the x-axis. “*” indicates differences between all lower groups when compared with all upper groups (*p* < 0.05). “✦” indicates significance with WT upper and PASK lower clusters only, “✤” is PASK upper with PASK lower only, while “✩” indicates the PASK lower cluster mice are significant with respect to all other cohorts (*p* < 0.05). (**B**) Four phyla displayed differences between the WT and PASK upper and lower cluster cohorts. “*” indicates *p*-value < 0.05. Factorial ANOVA in JMP Pro 14 was utilized followed by post hoc Tukey HSD analysis to determine significant differences between cohorts.

The examination of the two 22-week HFHS clusters revealed significantly higher levels of several phyla in the lower HFHS relative to the upper, including Bacteroidetes, Firmicutes, Actinobacteria, Proteobacteria, Saccaribacteria, Defferibacteres, Tenericutes, and an uncharacterized bacterium (*p* < 0.005, Figure 5). Factorial analysis revealed these relative differences to be due to an interaction between diet and genotype for each phyla, with some significant findings (Figure 5). Most phyla split into two significant groups by upper and lower cluster, displaying significant differences between all the upper and lower clusters, namely, the Bacteroidetes (*p*-values < 0.0031 for all combinations of upper and lower clusters), Firmicutes (*p*-values < 0.0001), Actinobacteria (*p*-values < 0.0048), and the Saccaribacteria (*p*-values < 0.001), suggesting the contributions due to genotype are subtle. However, the other phyla display more apparent genotypic effects. The Proteobacteria in the PASK lower cluster mice were significantly different from all other groups (*p*-value with PASK upper = 0.0002, WT upper = 0.0039 and WT lower = 0.0135), while Proteobacteria in the WT lower cluster were not different from any upper cluster group (PASK upper *p* = 0.1216 and WT upper = 0.7450). Defferibacteres displayed significance between the PASK lower and WT upper only (*p* = 0.0239), with less, but likely, significance with the PASK upper (*p* = 0.0705). The *p*-values of all the combinations with WT lower were >0.189, suggesting it was not significant. Tenericutes also displayed genotypic effects associated with the PASK lower cluster only (*p* = 0.0187 with PASK upper). The undefined phyla related to cyanobacteria displayed significance between only the PASK lower and upper clusters (*p* = 0.0102), with the WT lower cluster approaching significance with the PASK upper (*p* = 0.0732). These four phyla appear to be more associated with genotype, and their association may help explain some of the PASK-dependent phenotypes, including resistance to triglyceride accumulation on the HFHS diet. We, therefore, investigated the weight, triglyceride, GTT, and ITT data, with respect to these “upper” and “lower” clusters.

### 3.5. Microbiome Composition Is Associated with Glucose Tolerance in Mice on an HFHS Diet

**Figure 6:**
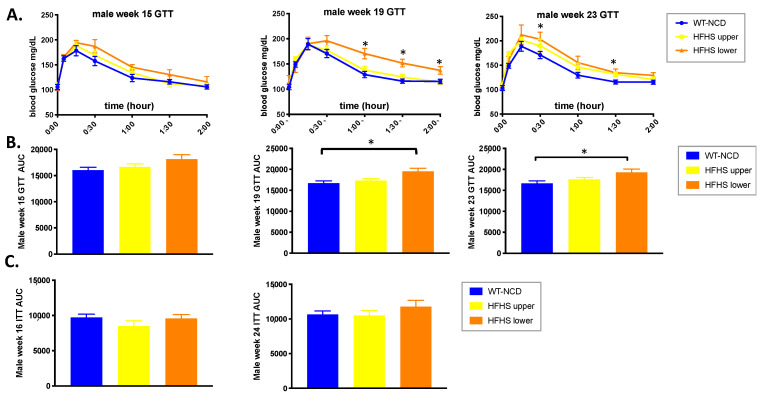
Gut microbial composition influences glucose response. (**A**) Week 15, 19 and 23 glucose tolerance test (GTT). (**B**) Area under the curve (AUC) of the GTT shown in (**A**) (*n* = 8–14). (**C**) Week 16 and 24 insulin tolerance test (ITT) (*n* = 10–13). ANOVA in JMP Pro version 14 was used to analyze significance with post hoc Dunnett’s analysis to determine significance with respect to the WT-NCD (* indicates *p* < 0.05).

The assignments of mice to the two clusters (upper and lower) observed at week 22 were first used to reanalyze GTT data from weeks 15, 19 and 23 by cluster, for microbial associations. This analysis revealed no difference between the clusters at week 15, but a shift to impaired glucose response was detected in the lower cluster, but not the upper cluster at week 19 (*p* = 0.0139 relative to NCD and 0.0366 relative to upper) and week 23 (*p* = 0.0099 relative to NCD, while upper was 0.4144 relative to NCD) (Figure 6A,B), which was consistent with the higher final body weights observed in the lower cluster (Figure 4C). These results are also consistent with the microbiome shift observed at week 22, but not observed at week 15 (Figure 4A). In contrast to this association of GTT with gut microbiota, the ITT results were not correlated with the microbiota composition (Figure 6C).

### 3.6. Genotype Influences Bacterial Composition, and Their Interplay Is Associated with Glucose Response in HFHS Mice

**Figure 7:**
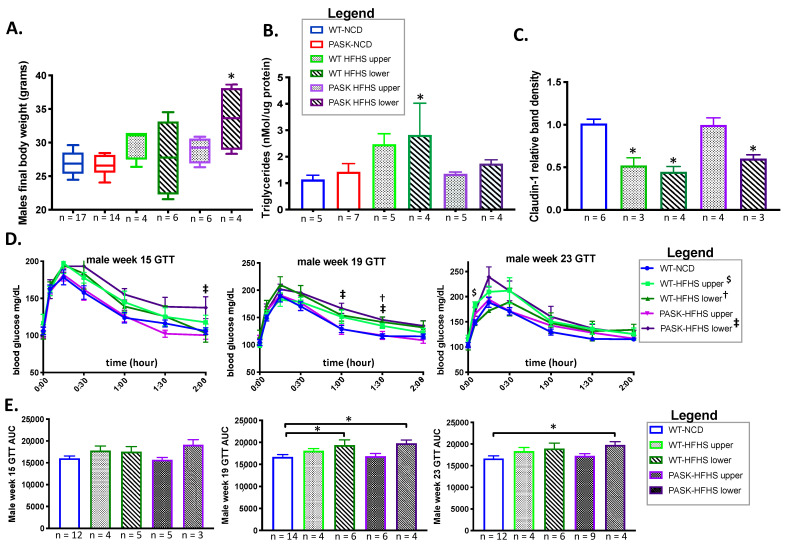
An interplay between genotype and microbial composition influences weight and glucose response. (**A**) Comparison of weight gain, (**B**) triglyceride accumulation, and (**C**) claudin-1 expression on the high-fat high-sugar diet by unweighted UniFrac PCoA cluster and genotype with sample number provided on the x-axis. (**D**) Glucose tolerance (GTT) tests at week 15, 19 and 23. (**E**) Area under the curve (AUC) of the GTT’s shown in (**D**), with sample number provided on the x-axis. ANOVA followed by post hoc Dunnett’s analysis using WT-NCD as control was performed on all samples, including individual GTT time points (symbols †, ‡, $ for *p* < 0.051) as well as the AUC (* is used for *p* < 0.051) using JMP Pro version 14.

To determine whether genotype played any role in the gut microbiota divergence and associated phenotypes, the upper and lower clusters were compared by genotype to the WT-NCD mice, for weight gain, liver triglyceride accumulation, claudin-1 expression, and glucose tolerance. When separated by genotype and microbiome cluster, an HFHS diet significantly increased the final body weight of the PASK-HFHS lower, but not upper, cluster mice, which is consistent with the trends toward higher body weight in the male PASK mice, suggesting an association (see Figure 1A). In contrast, the WT-HFHS lower cluster mice showed a significant increase (*p* = 0.0500) in triglycerides, relative to WT-NCD, with the WT-HFHS upper mice nearing significance (*p* = 0.0622), while both the PASK upper and lower clusters appeared protected (*p*-values = 0.9937 and 0.0822, respectively) (Figure 7). These results are consistent with altered lipidomics PASK^−/−^ mice, favoring triglyceride accumulation for WT mice and fat production for PASK^−/−^ mice, in response to the HFHS diet (Figure 1), and suggest microbial associations with WT triglyceride accumulation on an HFHS diet.

The HFHS diet decreased claudin-1 expression in both WT upper (*p*-value = 0.0025) and lower (*p* = 0.0002), as well as the PASK lower group (*p* = 0.0067), when compared to WT-NCD (Figure 7C). However, the PASK-HFHS upper group was protected and displayed claudin-1 levels similar to WT-NCD (*p* = 0.9995), just as this cohort was protected from weight gain (see Figure 7A). The factorial ANOVA performed previously (Section 3.1) suggested these effects were due to an interaction between genotype and diet, supporting the differences between WT and PASK, and suggesting a protective effect provided by PASK deficiency, which could be overcome by the microbiota in the lower cluster, but not the upper cluster. Factorial ANOVA was not used in this analysis, due to the lack of corresponding upper or lower clusters in NCD mice.

The glucose response by genotype in the upper and lower clusters was also compared to the WT-NCD mice (Figure 7D,E). At 15 weeks, there were no AUC differences that achieved statistical significance, by genotype, between the groups. However, the final datapoint of the GTT was significant for the PASK-HFHS lower group (*p* = 0.0552), and the AUC trended high (*p* = 0.0885). At 19 weeks, both the PASK-HFHS lower group (*p* = 0.0403) and the WT-HFHS lower group (*p* = 0.0371) had greater AUC. At the 23-week time point, only the PASK-HFHS lower group was significantly different from the WT-NCD group (*p* = 0.0534), with the WT-HFHS differences becoming less apparent (*p* = 0.1193). These results support both the microbial and genotypic effect suggested by the earlier factorial ANOVAs. The PASK-HFHS lower cluster mice showed a greater susceptibility to impaired glucose response, with the shift in the microbiota showing more dramatic impairment than WT-HFHS at weeks 15 and 23.

## 4. Discussion

Rates of obesity, hypertriglyceridemia, and insulin resistance are alarming, and are on the rise in the United States, necessitating improved animal models to better understand influencing factors, including the microbiome. The deletion of PAS kinase (PASK) has previously been shown to protect male mice against high-fat diet-induced weight gain, triglyceride accumulation, and insulin resistance [28,29,38], suggesting it is a key regulator of these pivotal pathways, yet no studies have investigated the combined effects of the microbiome and PASK deficiency. Herein, the role of PAS kinase was examined on a western-style HFHS diet, including effects on weight gain, triglyceride accumulation, glucose sensitivity, and insulin resistance, as well as microbiome composition, allowing us to investigate genotype, diet and microbiome interactions.

The previously reported protection from weight gain on a high-fat diet, conferred by PASK^−/−^ [28], was not observed in this study of the western HFHS diet. In fact, both male and female weights on the HFHS diet trended higher, and were significant at several time points, when the WT mice were not (see Figure 1A). Male mice were, however, protected from hepatic triglyceride accumulation as was observed previously [28], as well as decreases in claudin-1, a protein pivotal to the formation of leaky gut and inflammation. Herein, we also report the first evidence for dyslipidemia in female PASK^−/−^ mice, which had increased RFP on the HFHS diet (see Figure 1C). These differences in PASK^−/−^ mice were attributed to an interaction between diet and genotype, by factorial ANOVA.

The microbiome was analyzed at week 15 and week 22, which were time points chosen for maximum GTT/ITT difference (15 weeks, Figure 2), maximum length of time on the diet, and longest amount of time after blood glucose testing (2 weeks), to minimize any effects of stress. Although all mice displayed an expected shift to lower alpha diversity and increased beta diversity on the HFHS diet, a key finding was the remarkable forked separation of microbiome composition observed in both wild-type and PASK^−/−^ mice on the HFHS diet. Two discrete microbiomes emerged, with the “lower” cluster containing elevated levels of several bacterial phyla, including Firmicutes, Bacteroidetes, Actinobacteria, Proteobacteria, Saccharibacteria, Defferibacteres, and Tenericutes, when compared to the “upper” cluster (Figure 5). This unexpected finding altered the focus of our study to include the investigation of the effects of this split in microbiome composition. The upper and lower clusters were formed independent of genotype, and were associated with key differences in weight gain, liver triglyceride accumulation, and glucose intolerance.

An examination of the upper and lower microbiota clusters revealed that none of the mice in the upper cluster were in the highest tertile of body weight, suggesting the gut microbiota represented in that cluster might play a protective role in weight gain, or, alternatively, that the gut microbiota represented in the lower cluster might make mice more vulnerable to weight gain. When the upper and lower clusters were examined by genotype, only the PASK-HFHS lower cluster mice displayed a significant increase in body weight compared with the WT-NCD control. Thus, PASK-HFHS likely contributed the most to the weight gain observed, which is in opposition with the reported protection from weight gain observed for male PASK^−/−^ mice on a high-fat diet [28]. In contrast, when analyzing liver triglyceride by cluster, the WT lower cluster displayed significant increases, but the PASK^−/−^ mice appeared protected, supporting PASK deficiency protection from liver triglyceride accumulation (Figure 7A,B), as has been previously reported for PASK^−/−^ mice on both the high-fat [28] and HFHS diets [31]. In fact, the most dramatic phenotype observed for PASK^−/−^ in previous studies is the protection from liver triglyceride accumulation [28,31], explaining why genotype (PASK^−/−^) is able to overcome diet in this case. Both weight and liver triglyceride accumulation are associated with lower cluster microbiome on the HFHS diet, with key differences due to the genotype, including WT and PASK^−/−^ mice favoring liver triglyceride accumulation or body weight gain, respectively. These differences in body weight and liver triglyceride levels are likely due to the reported molecular function of PAS kinase in the regulation of SREBP-1c maturation and function, a key transcription factor for hepatic triglyceride biosynthesis [32,55]. PAS kinase has also been shown to regulate lipids through the direct phosphorylation and inhibition of Cbf1 in yeast, the homolog of human USF1 and a key fatty acid transcription factor [33,56].

In addition to increased total weight and triglycerides, the mice in the lower microbiota cluster also displayed significantly poorer glucose tolerance after both 19 and 23 weeks on the HFHS diet, whereas the mice in the upper microbiota cluster, on the same diet, showed a glucose response that was indistinguishable from that of wild-type mice on a healthy NCD (Figure 6A,B). When analyzed by genotype, the WT and PASK lower cluster mice both showed poor glucose tolerance (AUC) at 19 weeks, with only PASK AUC remaining significant at 23 weeks (Figure 7E). These differences, due to genotype, are consistent with the full factorial ANOVA results, which suggested that the effects on glucose tolerance were due to an interaction between diet and genotype. Taken together, the effects on glucose tolerance observed in this study appear to be primarily due to diet, with significant contributions from genotype. Although the diet effects on glucose tolerance are significant, but not dramatic, they are supported by previous studies of PASK^−/−^ mice, which report the dysregulation of genes involved in glucose sensing ([30]), insulin gene expression [28,29,30,36,37,41] and glucagon production [36,41], as well as human mutations that lead to insulin resistance [42].

The exact mechanisms by which gut bacteria influence weight gain, insulin resistance and hepatic triglyceride levels are unknown, but may be related to the inflammatory response triggered by lipopolysaccharides (LPS), which are endotoxins from the cell walls of Gram-negative bacteria. Dietary sugar has been shown to lead to increased hepatic fat and translocation of LPS from the intestines into the bloodstream of mice [57]. This circulating LPS is also correlated with obesity and insulin resistance [16,17]. LPS can pass through the intestinal epithelium into the bloodstream when tight junctions between epithelial cells are disrupted, often through a decrease in tight junction proteins, such as claudin-1 [27]. Current research suggests that gut microbiota influences the levels of these tight junction proteins via the protein kinase B (Akt) signaling pathway. LPS from Gram-negative bacteria binds to Toll-like receptor 4 on the surface of a cell, which triggers the production of inflammatory cytokines, including tumor necrosis factor α (TNF-α) and NFκB [58,59]. In intestinal epithelial cells, NFκB activates inflammatory cytokines, which inhibit the Akt/mTOR signaling pathway, reducing the levels of tight junction protein expression [60,61]. The WT-HFHS mice in our study displayed significantly less claudin-1 in the colon, whereas the PASK^−/−^ mice were protected against an HFHS-induced claudin-1 decrease (see Figure 1F and Figure 7C). Decreased levels of tight junction proteins in the gut have been shown to correlate with increased gut permeability, systemic inflammation, and insulin resistance [16,17]. Thus, changes in claudin-1 expression may in part explain the differences in glucose tolerance reported herein, along with the previously described regulation of insulin production and secretion by PAS kinase [34,35].

Even though the PASK^−/−^ mice were not protected against all of the harmful effects of the HFHS diet in this study (weight gain in the lower cluster), they did show some metabolic advantages over the wild-type mice. These primarily include overall resistance to triglyceride accumulation and increased claudin-1 expression in the upper cluster, as well as minor effects on glucose tolerance. Thus, overall, the factorial ANOVA analysis performed in this study suggests that the phenotypic effects are due to an interaction between genotype, diet and microbiome composition, supporting the differences between WT and PASK^−/−^, and suggesting a protective effect provided by PASK deficiency, which could be overcome by the microbiota in the lower cluster in the case of claudin-1 expression, weight gain, or glucose intolerance, but not triglyceride accumulation.

Increases in several key bacterial phyla were identified in this study, and were associated with the lower microbial cluster, which was most likely associated with the shift in the HFHS beta diversity observed at 22 weeks (see Figure 3). Factorial ANOVA suggested these increases were due to interactions between diet and genotype. Analysis of nine of these key phyla revealed five that were increased in both the WT and PASK lower cluster mice, with larger increases in the PASK mice (Figure 5). Three additional phyla displayed significant increases in PASK compared with the WT mice, namely, Proteobacteria, Defferibacteres, and Tenericutes. These associated bacteria may interplay with other phenotypic effects of PAS kinase deficiency, potentially influencing weight gain, triglyceride accumulation, and glucose sensitivity.

The cause of the divergence in microbiota composition between the upper and lower clusters is unknown. Maternal influences are a possible source of such differences, but the similarity of the HFHS microbiota at 15 weeks makes maternal influences a less likely explanation. Housing effects can also contribute to such differences, but because both the upper and lower clusters contained a mix of wild-type and PASK^−/−^ mice, and the two genotypes were housed separately, this explanation also seems less likely. It is possible that the microbiota shift happened in some mice due to stress, which is a known disrupter of the gut microbiota [62] and of tight junction protein expression [61,63].

Regardless of the cause of the divergence in gut microbiota composition, a key observation of this study is that the divergence only occurred in mice on the HFHS diet. No gut microbiota divergence was detected in the mice on the healthy NCD. This finding suggests the possibility that the healthy diet conferred protection against potentially harmful disruptions to the gut microbiota, whereas the western-style diet left mice vulnerable to such disruption, laying the foundation for further study.

## Data Availability

The data presented in this study are available within this article entitled “Gut Microbiota Regulates the Interaction between Diet and Genetics to Influence Glucose Tolerance”.
